# DNA profiling with the 20K apple SNP array reveals *Malus domestica* hybridization and admixture in *M. sieversii*, *M. orientalis*, and *M. sylvestris* genebank accessions

**DOI:** 10.3389/fpls.2022.1015658

**Published:** 2022-10-13

**Authors:** Gayle M. Volk, Cameron P. Peace, Adam D. Henk, Nicholas P. Howard

**Affiliations:** ^1^ United States Department of Agriculture-Agricultural Research Service (USDA-ARS) National Laboratory for Genetic Resources Preservation, Fort Collins, CO, United States; ^2^ Department of Horticulture, Washington State University, Pullman, WA, United States; ^3^ Fresh Forward Breeding and Marketing B.V., Huissen, Netherlands

**Keywords:** crop wild relatives, cultivar, genetic diversity, genotype, Central Asia

## Abstract

The USDA-ARS National Plant Germplasm System (NPGS) apple collection in Geneva, NY, USA maintains accessions of the primary *Malus domestica* (Suckow) Borkh. progenitor species *M. sieversii* (Ledeb.) M. Roem., *M. orientalis* Uglitzk., and *M. sylvestris* (L.) Mill. Many of these accessions originated from seeds that were collected from wild populations in the species’ centers of diversity. Some of these accessions have fruit phenotypes that suggest recent *M. domestica* hybridization, which if true would represent crop contamination of wild species populations and mislabeled species status of NPGS accessions. Pedigree connections and admixture between *M. domestica* and its progenitor species can be readily identified with apple SNP array data, despite such arrays not being designed for these purposes. To investigate species purity, most (463 accessions) of the NPGS accessions labeled as these three progenitor species were genotyped using the 20K apple SNP array. DNA profiles obtained were compared with a dataset of more than 5000 unique *M. domestica* apple cultivars. Only 212 accessions (151 *M. sieversii*, 26 *M. orientalis*, and 35 *M. sylvestris*) were identified as “pure” species representatives because their DNA profiles did not exhibit genotypic signatures of recent hybridization with *M. domestica*. Twenty-one accessions (17 *M. sieversii*, 1 *M. orientalis*, and 3 *M. sylvestris*) previously labeled as wild species were instead fully *M. domestica*. Previously unrealized hybridization and admixture between wild species and *M. domestica* was identified in 230 accessions (215 *M. sieversii*, 9 *M. orientalis*, and 6 *M. sylvestris*). Among these species-mislabeled accessions, ‘Alexander’, ‘Gold Reinette’, ‘Charlamoff’, ‘Rosmarina Bianca’, and ‘King of the Pippins’ were the most frequently detected *M. domestica* parents or grandparents. These results have implications for collection management, including germplasm distribution, and might affect conclusions of previous research focused on these three progenitor species in the NPGS apple collection. Specifically, accessions received from the NPGS for breeding and genomics, genetics, and evolutionary biology research might not be truly representative of their previously assigned species.

## 1 Introduction

Apple (*Malus*) genebank collections make plant genetic resources available to research and breeding programs that seek new alleles for improving disease and pest resistance, reducing environmental vulnerabilities, and improving production and consumer traits (Volk et al., 2015a; Bramel and Volk, 2019; Peace et al., 2019). Genebanks include *Malus domestica* cultivars as well as accessions that represent diverse species. For breeding, closely related *Malus* crop wild relatives provide desirable alleles without the extreme challenges of working with more distant wild species (Migicovsky and Myles, 2017). Researchers and breeders depend on apple collections to provide high quality materials that are true-to-type, at both the species and cultivar levels.

Donations, exchanges, and plant explorations have made the USDA National Plant Germplasm System (NPGS) apple collection among the largest and most diverse collections in the world (Volk et al., 2015a; Bramel and Volk, 2019; Gutierrez et al., 2020). The collection provides a wide range of *Malus* crop wild relatives that have been used to determine evolutionary relationships, identify novel alleles, and assess genetic diversity (Volk et al., 2015a). These crop wild relatives, including the primary *M. domestica* progenitor species of *M. sieversii*, *M. sylvestris*, and *M. orientalis*, were acquired by the NPGS through plant exchange and exploration expeditions that were performed between 1989 and 2004 (Luby et al., 2001; Forsline et al, 2002; Volk et al., 2009b). Exploration efforts introduced either budwood or seeds into the NPGS after passing through the United States national quarantine program. Budwood was grafted onto rootstocks and some seedlots were planted to obtain seedling trees in orchard blocks in Geneva, NY. Many of the trees have been genotyped using a set of 7 or 19 microsatellite markers, and the results were used to identify core subsets and genetic relationships among accessions (Richards et al., 2009a; Richards et al., 2009b; Volk et al., 2005; Volk et al., 2009b). Core subset and elite accessions (those exhibiting unusual or desirable phenotypes) were propagated by grafting and included in the permanent orchard collection. In addition, seedling trees produced from crosses between ‘Royal Gala’ and *M. sieversii*-labeled accessions PI 613971 (GMAL 4327), PI 613981 (GMAL 4448), and PI 613988 (GMAL 4455) resulted in research populations with local identifiers GMAL 4590, GMAL 4593, and GMAL 4595, respectively. These populations have been used for multiple genetic linkage mapping studies involving the *Ma* locus influencing fruit acidity (Xu et al., 2012), resistance to blue mold (Norelli et al., 2014; Norelli et al., 2017), and resistance to apple scab (Wang et al., 2012).

*Malus sieversii*, native to Central Asia and Western China, offers novel allelic diversity for a plethora of traits that are important to breeding programs (Volk et al., 2015a; Liu et al., 2021). A primary progenitor species of *M. domestica*, *M. sieversii* has been the target of numerous studies that have focused either on materials from China or Central Asia (Zhang et al., 2007). The extensive NPGS collection of this species in Geneva, NY, was obtained by Phil Forsline, Herb Aldwinckle, A. D. Dzhangaliev, and their collaborators in Kazakhstan and other Central Asian countries in 1989, 1993, 1995, and 1996. Four collection trips resulted in a total of 894 seedlots, and hundreds of these seeds were planted in the Geneva orchards, with many others provided to collaborators both within the U.S. and abroad (Forsline et al., 2002). Some trees in the wild in Kazakhstan had fruit quality phenotypes that rivaled those of cultivars and were therefore considered “elites” during the 1990s collection trips. Johann Sievers, after whom *M. sieversii* was named, during his travels in 1790 described wild apples of Kazakhstan (near Ust-Kamenogorsk, 500 km northeast of “Site 9” and about 1100 km northeast of Almaty) as dwarf trees with apples the size of a chicken egg, having red and yellow cheeks, and that could be eaten from the trees (Nussenov, 2018). This suggests that, as early as 1790, larger wild *M. sieversii* apples were present in the Targabatai Mountains and other northeast regions of Kazakhstan. “Elites” and other trees were introduced into the NPGS as budwood and grafted onto rootstocks, while most *M. sieversii* accessions were introduced as seed. *M. sieversii* germplasm accessioned into the NPGS has been distributed as budwood of accessions resulting from imported budwood of wild trees considered “elites”, budwood of accessions grown from wild-collected seeds, and seed from crossing among wild accessions grown ex situ. These materials have subsequently been used to determine genetic relationships between *M. domestica* cultivars and *M. sieversii* (Robinson et al., 2001; Gharghani et al., 2009; Nikiforova et al., 2013; Duan et al., 2017; Wedger et al., 2021), to evaluate phenotypic diversity of traits (Janisiewicz et al., 2008; Fazio et al., 2009; Bassett et al., 2011; Jurick et al., 2011; Van Nocker et al., 2012; Fazio et al., 2014; Maguylo and Bassett, 2014; Harshman et al., 2017; Watts et al., 2021; Davies et al., 2022), and to identify QTLs and novel alleles (Xu et al., 2012; Wisniewski et al., 2020; Singh et al., 2021).

*Malus orientalis*, native to the Caucasus and Middle East, is also a likely contributor to the domesticated apple (Cornille et al., 2012; Cornille et al., 2014; Amirchakhmahgi et al., 2018). Its attributes of interest to breeding programs include late blooming, environmental adaptation, fire blight resistance (Amirchakhmaghi et al., 2022), and long-term storage (Khadivi et al., 2020; Moradi et al., 2022). *M. orientalis* in the NPGS was primarily collected in exploration trips to Turkey, Russia, Armenia, and the Republic of Georgia between 1998 and 2004 (Volk et al., 2009b). The NPGS accessions of *M. orientalis* have been used for genetic and phenotypic research (Gharghani et al., 2009; Volk et al., 2009b; Duan et al., 2017).

*Malus sylvestris*, found in localized wild populations throughout much of Europe, has increasingly become recognized as an important ancestral contributor to *M. domestica*, but has not been as extensively utilized in breeding and research as the other two main progenitor species (Cornille et al, 2012; Cornille et al., 2013; Duan et al., 2017). Much of the literature on *M. sylvestris* has instead focused on the issue of recent hybridization with *M. domestica* cultivars, which is reportedly rife in wild populations. Such hybridization has been identified in the East Ore Mountains of Germany (40% of trees being hybrids; Reim et al., 2013), Saxony (13% hybrids; Reim et al., 2020), the Rhine Valley (5% hybrids; Schnitzler et al., 2014), and the United Kingdom (30% hybrids or pure *M. domestica*; Ruhsam et al., 2019). A recent study identified seven of 115 *M. sylvestris* accessions to be admixed with *M. domestica* in The Netherlands’ field genebank collection (Buiteveld et al., 2021).

SNP arrays have become a very powerful tool in apple, particularly for use in haplotype-based analyses such as pedigree-based QTL analyses (Kostick and Luby, 2022), introgression tracking (Luo et al., 2020), and relatedness estimation (Howard et al., 2021a). Several SNP arrays have been developed for use in apple, with the Illumina Infinium^®^ 20K (Bianco et al., 2014) being the most common. While these SNP arrays were designed using panels consisting primarily of *M. domestica* cultivars without input from any wild *M. sieversii*, *M. orientalis*, or *M. sylvestris*, it is expected that they will work well with these species being the progenitors of domesticated apple and because the 20K SNP array was successfully used for introgression tracking of *M. sieversii* haplotypes (Luo et al., 2020).

Acknowledging the importance of providing true-to-type species materials in the NPGS apple collection, the purpose of this study was to provide accurate information about the extent of hybridization (clear single recent crossing events) and admixture (multi-generational species mixing without specific ancestors identified) in NPGS accessions of *M. sieversii*, *M. orientalis*, and *M. sylvestris*, using the Illumina Infinium 20K SNP array.

## 2 Materials and methods

### 2.1 Plant material

Leaves were sampled from a total of 383 *M. sieversii*, 36 *M. orientalis*, 44 *M. sylvestris*, and one *M. domestica*-labeled accessions from the USDA National Plant Germplasm System apple collection in Geneva, NY (**Table S1**; USDA, 2022).

### 2.2 DNA extraction

Fresh frozen (100 mg) or dried (50 mg) apple leaf tissue was pulverized to a fine powder and DNA extracted using a modified CTAB extraction procedure (File S1). DNA quality and quantity were determined using a spectrophotometer/fluorometer.

### 2.3 Genotypic analysis

Samples were genotyped on the Illumina Infinium^®^ 20K apple SNP array (Bianco et al., 2014). Raw SNP array data were curated according to Vanderzande et al. (2019). The resulting genome-wide SNP profiles for the accessions were added to a dataset of more than 5000 unique genotypic profiles sampled from 56 apple collections previously assembled for an ongoing collaborative apple pedigree reconstruction project (Howard et al., 2018). *Malus* unique genotype (MUNQ) codes used for the organization of duplicate genotypic profiles were provided *via* Denancé et al. (2020).

Admixture was identified *via* a combination of an analysis of Summed Potential Lengths or Shared Haplotypes (SPLoSH) information (Howard et al., 2021a) and Principal Components Analysis (PCA). The commonly used STRUCTURE analysis (Pritchard et al., 2000) was not used because our pilot study identified extensive pedigree structure between many wild accessions and extant domestic cultivars, because of the presence of extensive pedigree structure among *M. sieversii* accessions, because of the small number of *M. sylvestris* accessions available for analysis, because of the extensive pedigree structure inherent in any panel of domestic cultivars that could be included in a STRUCTURE analysis, and because of issues relating to the SNP inclusion bias on the 20K SNP array. These issues would have severely violated some of the assumptions made in the STRUCTURE model or would have otherwise resulted in unclear or misleading results.

Genetic duplicates and parent-offspring relationships were sought among the progenitor species accessions and the larger dataset of DNA profiles as described in Vanderzande et al. (2019). Close pedigree relationships and grandparent-grandchild relationships were identified using SPLoSH information as described in Howard et al. (2021a) using 20 cM as a threshold. This threshold was chosen to readily enable detection of any recent cultivar ancestors of the species accessions in the dataset. If one parent of an accession was identified, haplotype data deduced for the chromosomal homologs from the unknown second parent were also compared to the dataset to detect any likely recent cultivar ancestors.

Grandparent-grandchild relationships involving species accessions were considered likely present where the SPLoSH values between pairs were 512 cM or higher, representing at least 20% of the entire diploid genome [twice the 1280 cM haploid genetic length; Howard et al. (2021a)]. These thresholds were used instead of the estimated coefficient of relatedness models from Howard et al. (2021a) for three reasons. First, the coefficient of relatedness model estimates from Howard et al. (2021b) were made only using *M. domestica* cultivars that were expected to have a degree of haplotype sharing through multi-generational endogamy (*via* artificial selection). Such shared ancestry would be expected to inflate the estimated average SPLoSH values for grandparent-grandchild and half-sib relationships among *M. domestica* cultivars. Thus, in instances where species accessions had a *M. domestica* grandparent but otherwise appeared to be of a progenitor species origin, the SPLoSH value between them would not have that inflation and instead would more closely approximate the theoretical 25% of genome sharing for this relationship. Second, the 20-cM threshold used could have prevented detection of some real but shorter identical-by-descent haplotypes, leading to a total amount of genome-sharing less than the expected 25%. But a smaller threshold than 20 cM was not used because those could also have resulted in some false or artificially elongated shared haplotypes due to limitations of the SNP coverage (some gaps being present) and informativeness (undetected null alleles possible) of the 20K array. Third, using a genome-sharing threshold slightly less than 25% allowed for expected biological variation in proportion of genome inherited after two meioses. Thus, the minimum threshold of 512 cM used for likely grandparent-grandchild considered in this study was intended to address these points by limiting both the exclusion of real relationships and the inclusion of false relationships.

Accessions were classified as fully *M. domestica* if their entire pedigree consisted of *M. domestica* cultivars. Accessions were classified as hybrid if they had one *M. domestica* parent or at least one likely *M. domestica* grandparent. Accessions were classified as having a *M. domestica* component, and thus admixed, if they had SPLoSH values with any *M. domestica* cultivar of more than 10% of their genome (0.1 × 2 × 1280 cM = 256 cM) but could not be classified as hybrid or fully *M. domestica*. If accessions had SPLoSH values with numerous *M. domestica* cultivars that were between 7.5% and 10% of their genome, they were noted as such but not classified as having a *M. domestica* component. Progenitor species accessions lacking definitive or clear evidence of *M. domestica* introgression from SPLoSH information were compared to one another through PCA to identify outliers that could also indicate admixed individuals. PCA was conducted using prcomp in R (R Core team, 2022). SPLoSH information using 5 cM as a threshold was used as the input information instead of raw SNP data to diminish effects of unequal SNP informativeness across the material and chromosomes and to account for genetic linkage among SNPs. PCA results were used to confirm or clarify the recorded species of accessions without clear admixture by observing clustering patterns. Outlier accessions positioned outside but between the primary species clusters were noted as being possible hybrids/admixed between those species. Such accessions were also examined for any abnormally large SPLoSH values with *M. domestica* cultivars relative to those found in accessions that did not have outlier PCA positions to gain evidence for the possibility of smaller-scale admixture.

Some accessions were classified as having an “exotic” *Malus* component. Exotic *Malus* in this study refers to *Malus* species with fruit smaller than *M. orientalis*, *M. sieversii*, and *M. sylvestris* accessions and very long stems, such as *M. baccata* (L.) Borkh., *M. floribunda* Siebold ex Van Houtte, *M. × micromalus* Makino, and *M. toringo* (Siebold) de Vriese (also referred to as *M. sieboldii* Rehder), and which are all not primary progenitors of *M. domestica*. Accessions were considered as having an exotic *Malus* component if they shared more than 256 cM (i.e., at least 10% of the diploid genome) of SPLoSH using 20 cM as a threshold with a group of 11 phenotypically confirmed exotic *Malus* accessions (**Table S2**) that lacked significant SPLoSH values with *M. domestica* cultivars.

Passport details of 28 of the 44 *M. sylvestris* accessions were recorded in GRIN-Global (USDA, 2022; and confirmed by genotypic analyses here) as belonging to three separate full-sibling groups derived from four different parents, only two of which were available for genotyping. To prevent this pedigree structure from causing *M. sylvestris* accession outliers to appear in the PCA, SNP profiles for the two ungenotyped parents, ‘Oelsen 5’ and ‘Klipphausen’, were imputed using their recorded offspring *via* the method of Howard et al. (2021a) and the parents of these full-sib groups were used for PCA instead of the full-sibs.

### 2.4 Phenotypic analysis

Available fruit image data were downloaded from GRIN-Global for the 20K SNP array-genotyped NPGS accessions of *M. sieversii*, *M. orientalis*, and *M. sylvestris.* Additional photograph imaging (Nikon D7100, 4000 × 6000 pixels) of multiple fruit was conducted and then uploaded to GRIN-Global for *M. sieversii*, *M. orientalis*, and *M. sylvestris* genotyped accessions that did not previously have associated image data available. From the 234 images, phenotypic measurements were conducted for five fruit of each accession for the traits of fruit diameter, fruit ground color, percentage of fruits with overcolor, percentage of each fruit with red overcolor, and fruit shape (according to Watkins and Smith, 1997; **Figure S1**). Quantitative data were analyzed by ANOVA and Tukey Mean Separation tests.

## 3 Results

### 3.1 Genotypic analysis

**Figure 1:**
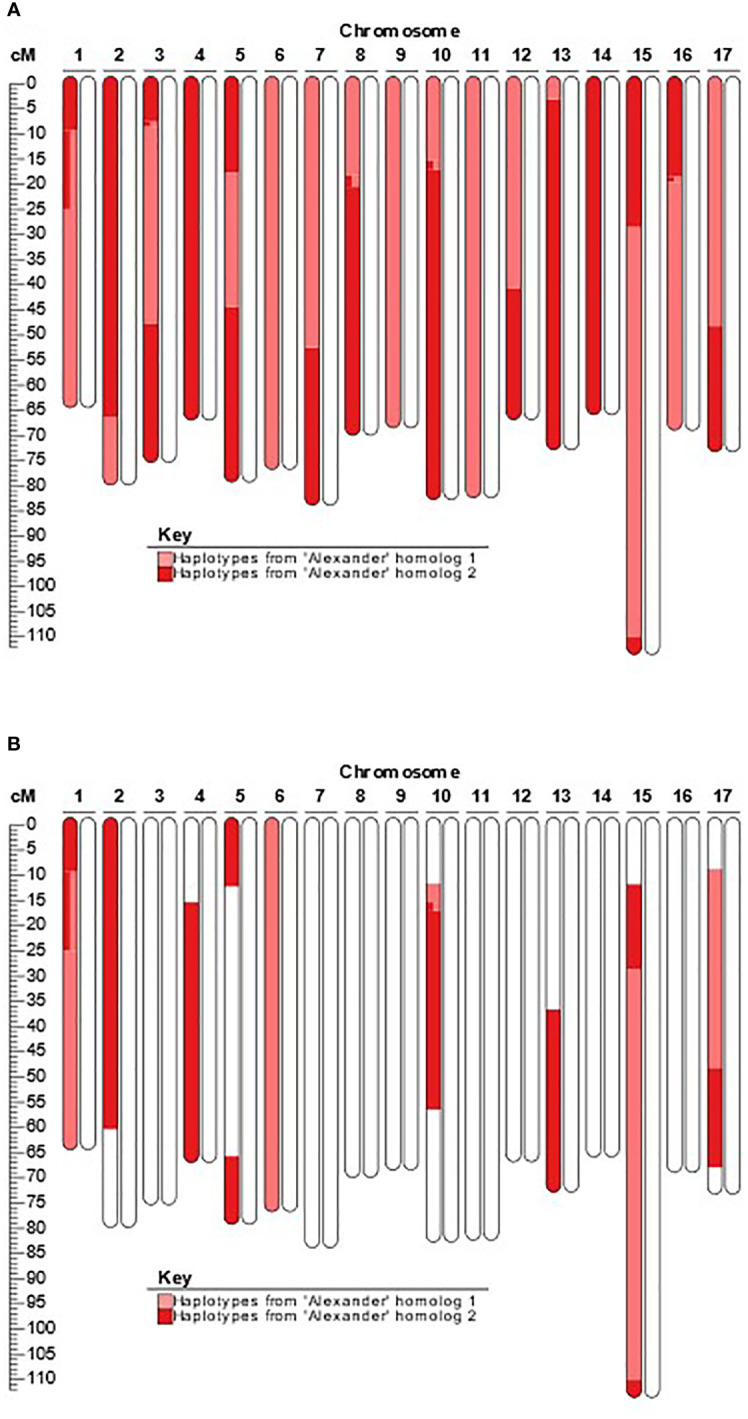
Example of newly detected presence of recent *M. domestica* ancestry in accessions previously labeled as pure wild progenitor species. **(A)**. Extended shared haplotypes of ‘Alexander’ present in the *M. domestica*-*M. sieversii *hybrid accession PI 613979; **(B)** Extended shared haplotypes of ‘Alexander’ present in the *M. domestica*-*M. sieversii *hybrid accession PI 650959.

The SNP array performed effectively on accessions of all three progenitor species to detect hybridization and admixture, enable pedigree reconstruction using SPLoSH information, and enable DNA profile imputation of two ungenotyped *M. sylvestris* parents. SPLoSH information was able to reliably illuminate clear instances of admixture, often directly through domestic cultivars. As an illustrative example, *M. domestica*-*M. sieversii* hybrid PI 613979 was identified as an offspring of ‘Alexander’ (**Figure 1A**), and phased haplotypes, with recombination evidence, from ‘Alexander’ clearly accounted for one homolog of each chromosome of PI 613979. In PI 650959, an offspring of PI 613979, remnant haplotypes of its grandparent ‘Alexander’ can clearly be identified (**Figure 1B**).

All accessions with a non-*M. domestica* component tended to have higher numbers of null alleles present than did pure *M. domestica* cultivars, although this did not seem to impede admixture detection. The *M. sieversii* accession with the highest level of data curation *via* descendants, PI 613981, had 41 SNPs detected as being homozygous for null alleles. For *M. orientalis*, the highest detected number of homozygous-null SNPs was 21 (in PI 682807), and the highest detected number in a curated *M. sylvestris* accession was 36 (in GMAL 4495.o, having SNP data available for both parents). PCA based on the SNP array data clearly differentiated the three progenitor species (**Figure S2**).

#### 3.1.1 *Malus sieversii*

Of the 383 accessions originally labeled as *M. sieversii*, 151 were determined to be pure *M. sieversii*, 17 were determined to be fully *M. domestica*, and 215 were hybrids or admixed between taxa. Specifically regarding the latter, 178 accessions were *M. sieversii-M. domestica* hybrids, 33 were *M. sieversii* with domestic components, one was a *M. sieversii-M. orientalis* hybrid, and three were *M. sieversii* with an exotic component (**Table S1**). In addition, one *M. domestica*-labeled accession (PI 644151) was determined to be a *M. sieversii-M. orientalis* hybrid. In all, only 39% of the sampled accessions labeled as *M. sieversii* in the NPGS genebank were determined to represent this species in its “pure” form.

Five pairs of accessions were identified with identical DNA profiles, all of which were originally labeled as *M. sieversii*. PI 657760 (DM 34) and PI 657763 (DM 49), both received from a Kyrgyz Republic exploration (Volk et al., 2009a), were identical. Duplicate accession pair PI 650966 and PI 650977 originated from a *M. sieversii* wild-collected seedlot(s) from Site 9 raised at the University of Minnesota and provided back to the NPGS apple collection. PI 614000 and PI 657764, collected from Kazakhstan Sites 9.02 and 9.04, respectively, were both identified as ‘Rosmarina Bianca’, an old Italian cultivar available from the United Kingdom’s National Fruit Collection. The duplicate pair of PI 613953 and PI 613978 was identified as *M. sieversii* with a domestic component. PI 613978 was collected in 1995 from Site 9.05, and a collection note by P. Forsline in 1996 suggested that PI 613953 might be the same tree. The duplicate pair of PI 657072 and PI 657117 was pure *M. sieversii*, although the two accessions were collected from different sites in Kazakhstan (Sites 6 and 12, respectively).

Of the thirty-six *M. sieversii*-labeled accessions classified as “elite” in the NPGS collection, five were determined to be *M. domestica*, 21 *M. sieversii-M. domestica* hybrids, three *M. sieversii* with a *M. domestica* component, and only seven were pure *M. sieversii*. The accession named ‘FORM 35’ (PI 613967), which was selected in Kazakhstan by Dr. Dzhangaliev and presumed to be *M. sieversii* (GRIN-Global, 2022), was determined to be a *M. sieversii-M. domestica* hybrid, with ‘Zigeunerin’ as one parent. ‘FORM 35’ was also determined to be a parent of two other “*M. sieversii*” accessions in the dataset (PI 629319 and PI 629318).

**Figure 2:**
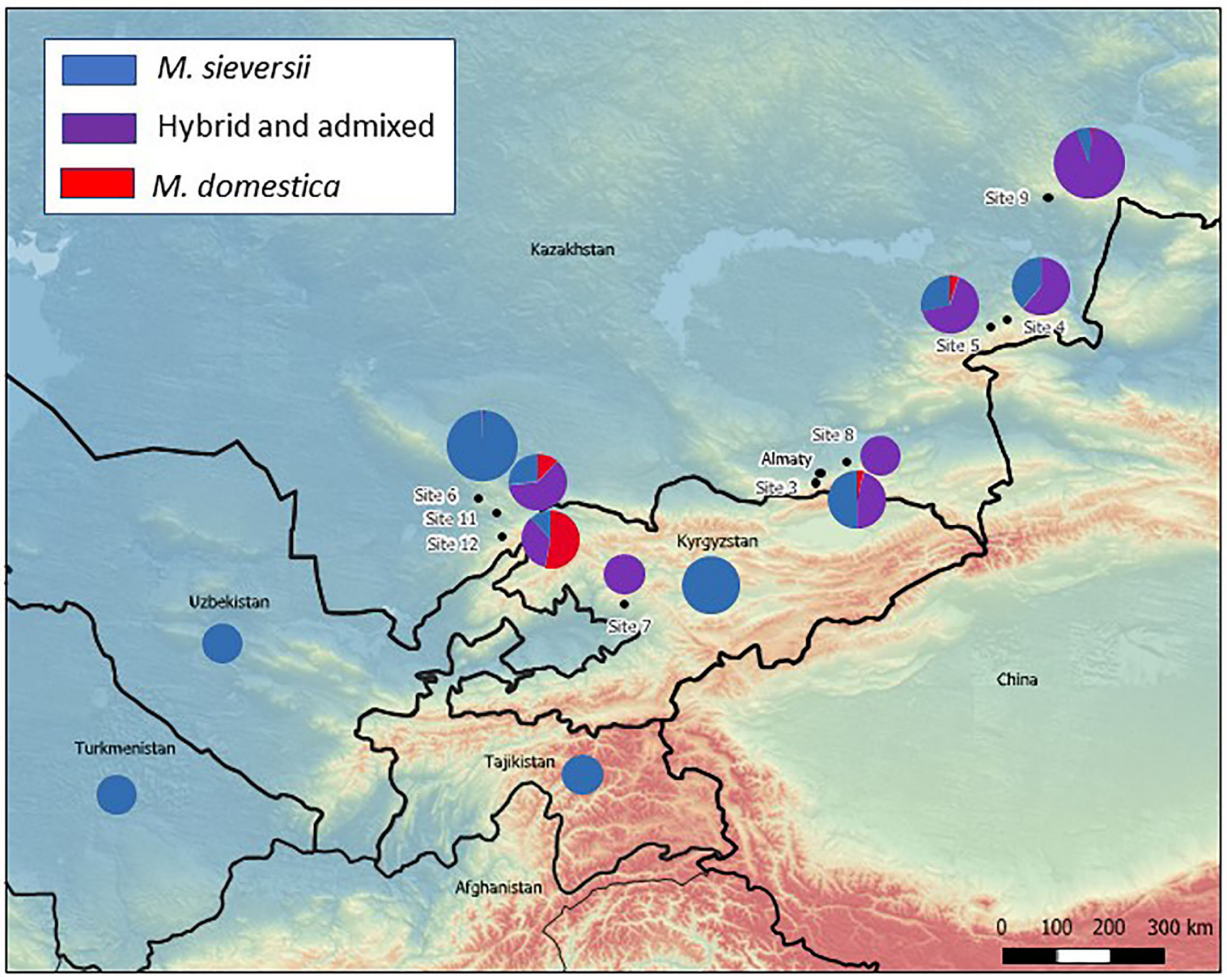
Map of *M. sieversii* collection sites for NPGS apple collection genotyped with a 20K SNP array. The subsequently determined proportions of pure *M. sieversii*, pure *M. domestica*, and hybrid/admixed individuals investigated from each site are overlaid as pie charts (small pie chart: 1-10 individuals; medium pie chart: 11-50 individuals; large pie chart: 51-135 individuals).

The extent of admixture in sampled populations varied across the original collection sites (**Figure 2**). All but one accession examined from Site 6, in the Karatau region, were determined to be all pure *M. sieversii*, but only 27% of the accessions of Site 11, also in the Karatau region, were pure *M. sieversii*. A large proportion of the accessions examined from Site 12 (53%) were *M. domestica* cultivars that had been originally considered to be *M. sieversii*. For the site with the largest representation in the dataset, Site 9, located in the Tarbagatai region, 93% of the tested individuals were identified as hybrid or admixed and only 6% were determined to be pure *M. sieversii* (**Table 1**; **Figure 2**). The majority (96%) of the 24 sampled accessions originating from outside of Kazakhstan were identified as pure *M. sieversii* (**Table 1**). Most (75%) of the 76 accessions from Kazakhstan-sourced seedlots that were provided to Dr. James Luby in 2007 that were grown, evaluated, selected among, and then returned to the NPGS were admixed with *M. domestica* (**Table S1**).

**Table 1:** Species compositions determined for NPGS *Malus sieversii* collection sites representing various regions in Central Asia.

Site/source (region)	Number of accessions (n)					Proportion of accessions (%)		
	Pure *M. sieversii*	Hybrid/admixed *M. sieversii*	Pure *M. domestica*	Total		Pure *M. sieversii*	Hybrid/admixed *M. sieversii*	Pure *M. domestica*
Site 1 (Tajikistan)	4	0	0	4		100	0	0
Site 2 (Uzbekistan)	6	0	0	6		100	0	0
Site 3 (Zailisky-Almaty, Kazakhstan)	14	13	1	28		50	46	4
Site 4 (Djungarsky-Topelevka, Kazakhstan)	7	11	0	18		39	61	0
Site 5 (Djungarsky-Lepsinsk, Kazakhstan)	12	28	2	42		29	67	5
Site 6 (Karatau, Kazakhstan)	74	1	0	75		99	1	0
Site 7 (Kyrgyzstan)	0	1	0	1		0	100	0
Site 8 (botanic garden, Kazakhstan)	0	1	0	1		0	100	0
Site 9 (Tarbagatai, Kazakhstan)	8	126	1	135		6	93	1
Site 11 (Karatau, Kazakhstan)	7	16	3	26		27	62	12
Site 12 (Talasky, Kazakhstan)	2	6	9	17		12	35	53
Diane Miller donation (Kyrgyzstan)	12	0	0	12		100	0	0
Donation (Turkmenistan)	1	0	0	1		100	0	0

Some *M. domestica* cultivars were repeatedly identified as parents and/or grandparents of “*M. sieversii*” accessions (**Tables 2**, **S1**, **S3**). Russian cultivars were identified as the most common source of *M. domestica* contamination in NPGS “*M. sieversii*” accessions collected directly from Tien Shan forests (Sites 3, 4, 5, 9, 11, 12; **Figure 2**; **Table S1**) and also as parents and grandparents of “*M. sieversii*” seedlings. ‘Alexander’, ‘Gold Reinette’, and ‘Charlamoff’ were the most prolific grandparents (**Table 2**). Some non-Russian cultivars were also found directly in the Kazakh landscape or as parents or grandparents of seedlings, such as ‘Rosmarina Bianca’, an old Italian cultivar (found twice at Site 12 and many times as a recent ancestor at Sites 11 and 12), ‘King of the Pippins’, originally from England, and cultivars from many other geographical origins.

**Table 2:** Named cultivar ancestors of NPGS apple accessions labeled as *Malus sieversii* (excluding genotypic duplicates) identified by pedigree reconstruction using SNP array genotypic information.

Cultivar or accession name	Number of accessions (n)	
	Parent	Grandparent
Anis Aliy	0	1
Charlamoff^1^	4	30
Cheal's Weeping	1	0
Duchess Favorite	0	1
Englische Spitalrenette	0	1
Form 35	3	0
Gold Reinette^1^	4	47
Grågylling	0	1
Alexander^3^	8	51
Kantil Sinap	2	0
King of the Pippins	7	10
Köstlicher	1	6
Kulon Kitaika	2	5
Landsberger Reinette^3^	1	0
Suislepper	5	2
Passe-Pomme Rouge	0	1
Reinette de Hollande^2^	1	7
Reinette Simirenko	2	0
Rosmarina Bianca	7	11
Red Astrachan	1	3
Saint Germain	0	3
Sipolins	0	4
Spasovka Kvasna	1	7
Yellow Bellflower	0	5
Yellow Transparent	0	6
Zigeunerin	2	13

^1^Charlamoff is a parent of Gold Reinette.

^2^Reinette de Hollande is a parent of King of the Pippins.

^3^Alexander is a parent of Landsberger Reinette.

#### 3.1.2 *Malus orientalis*

Admixture with *M. domestica* was observed in *M. orientalis* NPGS accessions to a lesser extent than in *M. sieversii* accessions. Twenty-six of the 36 accessions labeled as “*M. orientalis*” were pure *M. orientalis*, one (PI 644252) was determined to be fully *M. domestica* (an offspring of ‘Golden Delicious’ and ‘Delicious’), and nine were hybrid/admixed. Of the latter, three were *M. orientalis-M. domestica* hybrids (‘Delicious’ and ‘Eierapfel’ were parents), one was a *M. domestica*-exotic hybrid, four were *M. orientalis* with *M. domestica* components, one was a *M. orientalis-M. sieversii* hybrid, One of the *M. domestica-M. orientalis* hybrids, PI 682808.s, was a triploid that shared an allele at every locus with ‘Kasseler Renette’ (**Table S1**). In all, 72% of the *M. orientalis* accessions were identified as pure species representatives.

#### 3.1.3 *Malus sylvestris*

The least admixture was detected for *M. sylvestris* NPGS accessions. Of the 44 accessions labeled as *M. sylvestris*, 35 were pure *M. sylvestris*. All *M. sylvestris* accessions from the three half-sib groups had recorded pedigrees that were confirmed with 20K SNP array genotyping and enabled successful whole-genome imputation of the ungenotyped *M. sylvestris* parents ‘Oelsen 5’ (95.5% of all alleles) and ‘Klipphausen’ (99.8%). Three accessions labeled as *M. sylvestris* were *M. domestica*, three accessions were *M. sylvestris*-*M. domestica* hybrids and three were *M. sylvestris* with domestic components (**Table S1**). In all, 80% of *M. sylvestris* accessions were deemed pure species representatives.

### 3.2 Phenotypic analysis

**Figure 3:**
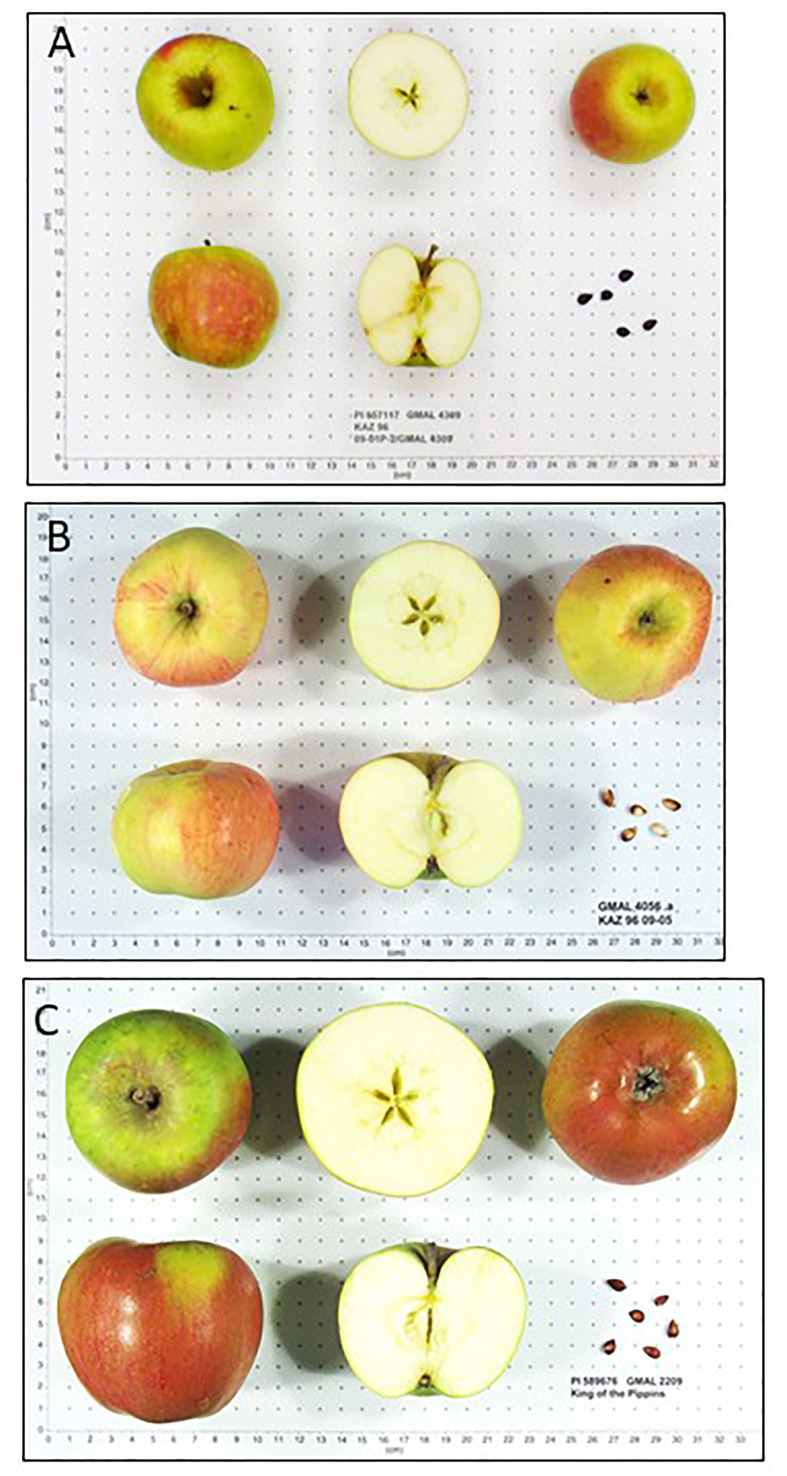
*Malus* fruit images from the USDA-NPGS apple collection **(A)** PI 657117 collected from Kazakhstan Site 12, determined to be pure *M. sieversii*; **(B)** PI 682787 collected from Kazakhstan Site 12, determined to be a hybrid between *M. sieversii* and *M. domestica ‘*King of the Pippins’; **(C)** Accession PI 589676, *M. domestica* ‘King of the Pippins’.

**Figure 4:**
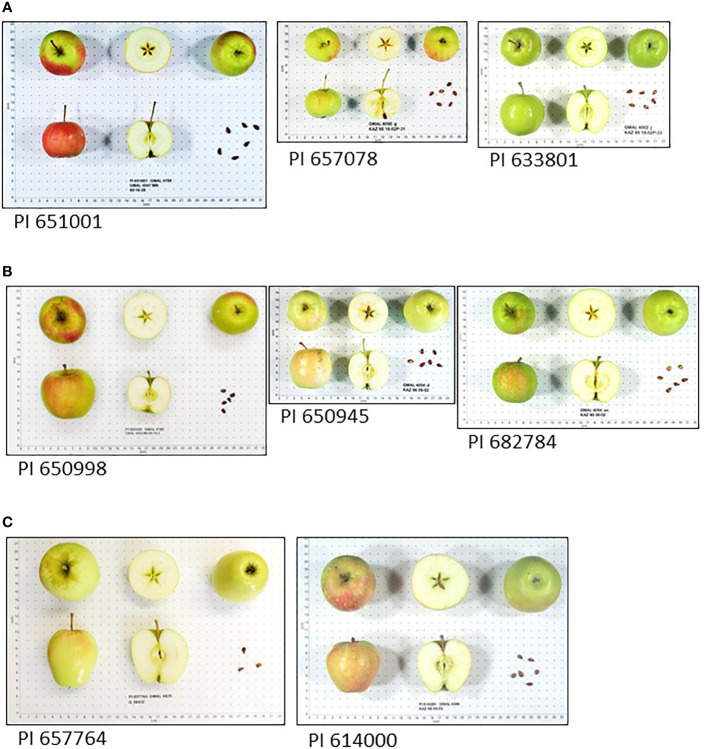
*Malus* fruit images from the USDA-NPGS apple collection **(A)** Three accessions collected in Kazakhstan determined to be pure *M. sieversii*; **(B)** Three accessions collected in Kazakhstan determined to be *M. sieversii-M. domestica* hybrids with ‘Rosmarina Bianca’ as a parent; **(C)** Two accessions collected as *M. sieversii* in Kazakhstan with genotype matching the United Kingdom National Fruit Collection accession 1951197, ‘Rosmarina Bianca’.

Fruit of *M. sieversii-M. domestica* hybrids and *M. orientalis-M. domestica* hybrids were significantly larger than those of their pure wild species counterparts (**Table 3**). There were too few *M. sylvestris-M. domestica* hybrids to establish that their fruit were significantly larger than those of pure *M. sylvestris* fruit (**Table 3**). Fruit ground color and proportion of red overcolor were generally similar among counterparts, considering that some fruit might have been sampled and imaged while immature. Pure *M. sieversii* and *M. sieversii-M. domestica* hybrids showed the greatest fruit shape diversity, while fruit of pure *M. orientalis* and *M. sylvestris* accessions were mostly globose and flat-globose (**Figures S3–S5**; **Tables 3**, **S4**). A series of image examples of the relationships between pure *M. sieversii* from Site 12, an *M. sieversii-M. domestica* ‘King of the Pippins’ hybrid from Site 12, and the cultivar ‘King of the Pippins’ are shown (**Figure 3**). In addition, a series of three pure *M. sieversii* accessions, three *M. sieversii-M. domestica* ‘Rosmarina Bianca’ hybrids, and two Kazakhstan-collected accessions that match genotypes of ‘Rosmarina Bianca’ are shown (**Figure 4**).

**Table 3:** Some fruit trait observations for pure and hybrid *M. sieversii*, *M. orientalis*, and *M. sylvestris* accessions in the USDA-National Plant Germplasm System apple collection.

Species classification *via* SNP analysis	n	Fruit diameter (cm)		Fruit ground color				Fruit overcolor		Fruit shape							
				green (%)	yellow (%)	red (%)		% Accessions with fruit exhibiting red overcolor	Average % of fruit surface with red overcolor	conical (%)	ellipsoid (%)	ellipsoid-conical (%)	flat (%)	flat-globose (%)	globose (%)	globose-conical (%)	oblong (%)
*M. sieversii*	87	4.63 ± 0.07 b		86	13	1		66	65	1	1	1	2	25	56	5	8
*M. orientalis*	18	3.38 ± 0.06 c		50	50			61	65				11	67	22		
*M. sylvestris*	15	2.94 ± 0.15 c		47	53			27	27					60	40		
*M. sieversii × M. orientalis*	1	4.96 abc		100				100	100						100		
*M. sieversii × M. domestica*	102	5.49 ± 0.11 a		63	36	1		75	75	3			3	28	58	2	6
*M. orientalis × M. domestica*	6	5.45 ± 0.57 ab		83	17			67	57					33	67		
*M. sylvestris × M. domestica*	5	4.62 ± 0.63 abc		60	20	20		80	100					80			20

## 4 Discussion

It is critical to have access to pure *M. sieversii*, *M. orientalis*, and *M. sylvestris* genebank materials because these species are recognized as valuable to and are being used in costly long-term breeding programs, assessments of domestication, and genetic dissection of traits of interest (Volk et al., 2015a). The extent of hybridization and admixture identified in this study between *M. domestica* and *M. sieversii*, *M. orientalis*, or *M. sylvestris* in the NPGS apple collection is significant. The identification of hybrid/admixed accessions in the NPGS apple collection presented herein may affect findings of research from around the world that used the collection to identify novel alleles and assess genetic relationships.

Pure cultivars and species-*M. domestica* hybrids were identified in NPGS accessions that were labeled as *M. sieversii*, *M. orientalis*, and *M. sylvestris*. In each case, *M. domestica* cultivars in the landscape could have been inadvertently sampled by plant exploration teams. In addition, hybrid trees may have grown from seeds derived from natural pollination occurring between wild species and locally grown *M. domestica* cultivars. Furthermore, collection teams may have sampled fruit from wild trees with seeds resulting from crosses with pollen from cultivars (or hybrids). The following exemplifies how trees in wild populations could have recent cultivar ancestors such as ‘Alexander’, which was detected as a common recent ancestor of many “*M. sieversii*” accessions in this study. ‘Alexander’, which was originally from Poland and brought to Kazakhstan in 1865 under the name of ‘Aport’, was a common cultivar in orchards in Kazakhstan until devastating freezes between 1951 and 1955 that destroyed most orchards. ‘Aport’ was then replanted within the *M. sieversii* forests (Barbera et al., 2016).

Our results revealed a greater extent of hybridization and admixture in the NPGS apple collection than previously described. Gross et al. (2012) used microsatellite marker genotyping to identify hybrids in the NPGS collection and reported that 10% of the sampled *M. sieversii* and *M. orientalis* and 20% of the sampled *M. sylvestris* was hybrid or admixed, as revealed by STRUCTURE results. Omasheva et al. (2017) reported the genotyping with microsatellite markers of 311 *M. sieversii* trees from 12 wild populations and 16 wild apple clones selected by Dzhangaliev and found the lowest levels of *M. domestica-M. sieversii* admixture in the Kazakh regions of Krutoe truct (89% pure *M. sieversii*) and Tauturgen (92% pure *M. sieversii*). These two sites are located near the present study’s Sites 5 (Djungarsky-Lepsinsk) and 3 (Zailisky-Almaty), respectively, where we detected high levels of hybridization/admixture. The difference could reflect the greater resolution of SNP arrays to detect hybridization/admixture compared to microsatellite marker systems or differences in localized areas of *M. domestica* contamination among sampled locations. The 16 Dzhangaliev clones sampled by Omasheva et al. (2017) were reported to all have some *M. domestica* admixture, which is similar to what we observed with the Dzhangaliev-selected accession ‘FORM 35’. Kazakhstan Site 6 had almost no detected admixture and could serve as a source of pure *M. sieversii* for reforestation purposes.

We found that the extent of *M. sieversii* admixture was greater in Kazakhstan than in other sampled Central Asian countries. Ha et al. (2021) also measured admixture of *M. sieversii* and *M. domestica* in Kazakhstan using microsatellite markers. That study sampled 84 *M. sieversii* trees from regions in the east that were near Sites 4 and 5 and towards the west near Site 3 of the current study. All of the populations sampled by Ha et al. (2021) exhibited some hybridization between *M. sieversii* and *M. domestica*, which is consistent with results in the present work for populations sampled from those locations. In contrast, the lower levels of detected admixture in *M. sieversii* sampled from outside of Kazakhstan (**Figure 1**) may reflect different cultural practices across the landscape resulting in fewer numbers of *M. domestica* trees in or nearby the native stands of *M. sieversii* in other Central Asian countries.

Core sets seek to capture the diversity of populations using a limited number of individuals; the current work has revealed that formerly proposed core sets included admixed individuals. The *M. sieversii* core sets were developed based on microsatellite genotypic data and fruit and disease resistance phenotypic data for individuals from Sites 6 and 9 in Kazakhstan (Volk et al., 2005). Site 6, which was mostly pure *M. sieversii* based on the SNP array analysis, had 31 of 35 core-set individuals that were pure *M. sieversii* and four that were not in the SNP array dataset. In contrast, Site 9, which exhibited high levels of *M. sieversii-M. domestica* admixture in the SNP array analysis, had only four pure *M. sieversii* accessions but 17 *M. sieversii-M. domestica* hybrids, two *M. sieversii* with *M. domestica* components, one *M. domestica*, and no data available for 11 of the 35 core set individuals. A third *M. sieversii* core set was proposed by Richards et al. (2009a) to represent NPGS *M. sieversii* individuals from other collection sites in Kazakhstan. Our results revealed that this set of 35 individuals had 12 pure *M. sieversii*, 19 *M. sieversii-M. domestica* hybrids, three *M. domestica*, and one with no data available. Therefore, the core collections based on Site 9 and the other Kazakhstan sites were identified here as containing a large extent of hybrids and admixture. While the *M. sieversii* core sets chosen by Volk et al. (2005) appear to be have captured the general degree of admixture that is present in the collection sites, the detected contamination with *M. domestica* indicates that these core sets should not be considered as representative of *M. sieversii* diversity there. In any case, each of the three core sets should contain enough representation by *M. sieversii* to enable the discovery of novel alleles.

The results revealed admixture in the individuals represented by the NPGS *M. orientalis* core set individuals proposed in Volk et al. (2009b). Fourteen of the 27 *M. orientalis* core set individuals overlapped with the current SNP dataset. Of those, nine were pure *M. orientalis*, one was a *M. orientalis-M. domestica* hybrid, three were *M. orientalis* with *M. domestica* components, and one was *M. sieversii* with *M. domestica* components. Pure *M. orientalis* was identified in trees that originated from Russia, Turkey, Armenia, and Georgia. Thus, despite contamination with other species, the *M. orientalis* core set appears to be mostly *M. orientalis* and should contain novel alleles. But it should not be considered as representative of *M. orientalis* diversity.

Previously reported phenotypic assessments performed using NPGS-derived materials are now identified as having unintentionally included hybrid and admixed accessions. Watts et al. (2021) provided phenotypic data for the Apple Biodiversity Collection in Nova Scotia, Canada, derived in part from NPGS accessions. The supplementary data in Watts et al. (2021) has 78 accessions labeled as *M. sieversii*, 55 of which were included in the present study. Of those, two were determined to be *M. domestica*, 21 were *M. sieversii-M. domestica* hybrids, one was *M. domestica* with an exotic component, one was *M. sieversii* with an exotic component, three were *M. sieversii* with *M. domestica* components, one was *M. sieversii* with possible admixture, and only 26 were pure *M. sieversii*. Davies et al. (2022) described the phenotypic divergence between *M. domestica* and *M. sieversii* using the same dataset. While that study revealed significant differences for traits including soluble solids content, bitterness, and firmness during storage between the trees labeled as *M. domestica* and those labeled as *M. sieversii*, those differences could be even more pronounced if only pure species accessions are considered.

The result of extensive *M. domestica* hybridization and admixture in *M. sieversii* accessions might have influenced previous conclusions of the implied species origins of interesting trait locus alleles. The mapping populations GMAL 4590 and GMAL 4595, used for fine-mapping of the *Ma* locus (Xu et al., 2012), were derived from “*M. sieversii*” parents PI 613971 and PI 613988, respectively, determined here to both be *M. sieversii-M. domestica* hybrids, with the *M. domestica* cultivar Charlamoff being a parent of PI 613971. Four of nine “*M. sieversii*” accessions with PI numbers that were phenotyped for fire blight responses in Washington and West Virginia and often exhibiting resistance (Harshman et al., 2017) were determined here to not be pure *M. sieversii*. Similarly, 21 misidentified and nine pure species PI accessions from the NPGS apple collection were tested for resistance levels to blue mold considering them to all be pure *M. sieversii* (Janisiewicz et al., 2008), and several accessions exhibiting moderate levels of resistance were hybrids and *M. domestica* in our results. Although Wedger et al. (2021) used NPGS apple collection materials that were considered pure according to previous work (Gross et al., 2012), seven of the 15 *M. sieversii* individuals that study sampled from the NPGS were not pure species representatives according to the present analysis. In contrast, mapping population GMAL 4593 was created with parent PI 613981 (Desnoues et al., 2018), a *M. sieversii* pure species representative. Accessions PI 613981 (*M. sieversii*) and PI 633825 (*M. sylvestris*) used by Sun et al. (2020) and Luo et al. (2020) were also pure species representatives. Although *M. sieversii*-*M. domestica* hybrid and admixed individuals were inadvertently used in previous studies for identifying alleles of interest, discovered novel alleles could still be valuable to breeding and research programs. In some cases, the use of these hybrid and admixed individuals in breeding programs might introduce alleles of interest while incorporating fewer disadvantageous attributes from wild species.

NPGS apple collection materials now determined to be hybrids rather than pure species have been used for whole genome sequence-based projects, which could affect the results and conclusions of those studies. For example, Velasco et al. (2010) used ten *M. sieversii* accessions to determine the relative distinction between *M. domestica* and *M. sieversii*, five of which were determined here to be misidentified (GMAL 4054.a and GMAL 4309.d – *M. domestica*; GMAL 3762.g, GMAL 4304.e, and GMAL 4309.c – *M. sieversii-M. domestica* hybrids). Among the 117 diverse *Malus* accessions used by Duan et al. (2017) to obtain genome sequence data, 10 of the 15 *M. sieversii* accessions from Kazakhstan, the only *M. orientalis*, and three of the seven *M. sylvestris* sampled from the NPGS apple collection were not the expected pure species representatives (while SNP array data are unavailable for a further one *M. sieversii* and three *M. sylvestris* in that study), which helps explain some of the unexpected population structure findings in that report. Migicovsky et al. (2021) also assessed genetic relationships among *M. sieversii*, *M. sylvestris*, and *M. domestica* accessions from the NPGS apple genebank collection, although the specific individuals used were not reported. Inclusion of non-pure accessions of these species would be expected to blur genetic relationships or even exacerbate differences.

Wild species accessions in the NPGS apple collection that were identified as hybrids or admixed here have been included in phylogenetic and wild-cultivar relationships investigations (in addition to the previously mentioned study of Duan et al., 2017). For a *Malus* phylogeny based on chloroplast sequencing, Nikiforova et al. (2013) used several NPGS accessions that were not pure *M. sieversii* as expected, but rather *M. sieversii-M. domestica* hybrids, including GMAL 3610, GMAL 3619, and GMAL 3638, as well as PI 594104, which is actually *M. domestica* with an exotic component. The inclusion of these materials might have affected results, particularly the close relationships detected between *M. sieversii* and *M. domestica*. Similarly, Volk et al. (2015b) inadvertently used some “*M. sieversii*” and “*M. sylvestris*” that were determined here to be pure *M. domestica*, *M. sieversii-M. domestica* hybrids, or *M. sieversii* with *M. domestica* components. Accessions of *M. domestica*, *M. sieversii*, *M. orientalis*, *M. sylvestris*, and other species were classified into three haplotype groups based on chloroplast sequences in which the four species could not be differentiated; however, the clarification of accessions species status here does not remedy the situation. Gharghani et al. (2009) relied on NPGS apple collection materials to determine relationships among old Iranian cultivars and wild *Malus* species. One of the two *M. sylvestris*, ten of the 19 *M. sieversii*, and one of the *M. orientalis* of that study were not pure species representatives according to our results.

The NPGS GRIN-Global database has already been partially updated to reflect revised species statuses as determined herein. This effort will continue and will ideally highlight which accessions labeled as *M. sieversii*, *M. orientalis*, and *M. sylvestris* are pure species representatives. This updated information should ensure that species identities for *M. sieversii*, *M. orientalis*, and *M. sylvestris* are correct in the NPGS apple collection. It is recommended that researchers using accessions of these three species received from the NPGS apple collection update species designations.

## Data availability statement

The data presented in the study are deposited in the Genome Database for Rosaceae, accession number tfGDR1063. The data are released and are available here: https://www.rosaceae.org/publication_datasets.

## Author contributions

GV, CP, and NH conceived the study. NH analyzed and interpreted data. AH performed laboratory analyses and visualized data. All authors wrote and reviewed the manuscript. All authors contributed to the article and approved the submitted version.

## Funding

This research was partially funded by the USDA National Institute of Food and Agriculture Hatch project 1014919, Crop Improvement and Sustainable Production Systems (WSU reference 00011) and by a 2019 USDA Apple Crop Germplasm Evaluation Grant.

## Acknowledgments

SNP data for “Eierapfel” (accession number 100011) was shared by the National Genebank for Plant Genetic Resources for Food and Agriculture (PGREL) in Switzerland. The use of trade, firm, or corporation names in this publication is for the information and convenience of the reader. Such use does not constitute an official endorsement or approval by the United States Department of Agriculture or the Agricultural Research Service of any product or service to the exclusion of others that may be suitable. USDA is an equal opportunity employer and provider.

## Conflict of interest

Author NH is employed by Fresh Forward Breeding and Marketing.

The remaining authors declare that the research was conducted in the absence of any commercial or financial relationships that could be construed as a potential conflict of interest.

## Publisher’s note

All claims expressed in this article are solely those of the authors and do not necessarily represent those of their affiliated organizations, or those of the publisher, the editors and the reviewers. Any product that may be evaluated in this article, or claim that may be made by its manufacturer, is not guaranteed or endorsed by the publisher.
