# Supplementary material for: DNA profiling with the 20K apple SNP array reveals Malus domestica hybridization and admixture in M. sieversii, M. orientalis, and M. sylvestris genebank accessions
Source: Front Plant Sci. 2022 Oct 13;13:1015658. doi: 10.3389/fpls.2022.1015658 (PMC9606829; doi:10.3389/fpls.2022.1015658)
Supplement: Supplementary file 1 [file DataSheet_1.docx]

**File S1: DNA extraction procedure**

Fresh frozen (100 mg) or dried (50 mg) apple leaf tissue was pulverized to a fine powder in 1.5 mL Qiagen (Germantown, MD) collection tubes with #3 steel shot using a Qiagen Tissue-Lyzer II, in 30 second bursts at 30 Hz. Tubes containing dried tissue were cooled on ice for 2 minutes between sessions of pulverization, while tubes containing fresh tissue were kept frozen in liquid nitrogen. Frozen tissue was allowed to warm for 2 min at room temperature, before 600 µl 65°C CTAB extraction buffer (see below) was added to all tubes, mixed well by inversion, and incubated in a rocking incubator at 60°C for 1 h. Solid material was pelleted by centrifugation at 6000 rpm (5800 rcf) for 10 minutes at room temperature in a Sigma 4-16S centrifuge (Osterode am Harz, Germany), and 500 µL liquid was transferred to fresh 1.5 ml tubes. RNAseA (5µL 20µg/mL; Invitrogen Purelink RNAseA cat no 12091021 (Waltham, MA) was added. The tubes were incubated, oriented horizontally, in a rocking incubator at 37°C for 20 min. 500 µL chloroform/IAA solution (see below) was added, and the tubes were briefly mixed by inversion prior to incubating at room temperature for 2 min. The aqueous phase was separated by centrifugation at 6000 rpm (5800 rcf) for 10 minutes, and 450 µL was transferred to a fresh 1.5 mL Qiagen collection tube. Cold isopropanol (315 µL) was added, and the tubes were well mixed by inversion then incubated at -20°C for 15 minutes to precipitate the DNA. The DNA was pelleted by centrifugation at 6000 rpm (5800 rcf) for 10 minutes, and the liquid quickly poured from the tubes. The tubes were drained inverted on paper towels for several minutes to remove as much of the liquid as possible. The DNA pellets were then rinsed with 1 mL -80°C 80% ethanol and the tubes were centrifuged at 6000 rpm (5800 rcf) for 5 minutes to re-attach any loosened DNA pellets. The liquid was quickly poured off and the tubes allowed to drain inverted on paper towels for several minutes until the DNA pellets were no longer shiny. The DNA was resuspended in 50 µL TE (see below) and incubated at 60° for 10 min. The 260/280 OD ratio was determined with a Denovix DS-11 FX+ spectrophotometer/fluorometer (Wilmington, DE).

CTAB extraction buffer

100 mM Tris HCl (pH 7.5)

25 mM EDTA

1.5 M NaCl

2% CTAB

1% Polyvinylpyrrolidone

0.3% 2-Mercaptoethanol (added just prior to use)

Chloroform/IAA solution

96% Chloroform

4% Isoamyl alcohol

TE

10 mM Tris-Cl (pH 8.0)

1 mM EDTA (pH 8.0)
