# Supplementary material for: DNA profiling with the 20K apple SNP array reveals Malus domestica hybridization and admixture in M. sieversii, M. orientalis, and M. sylvestris genebank accessions
Source: Front Plant Sci. 2022 Oct 13;13:1015658. doi: 10.3389/fpls.2022.1015658 (PMC9606829; doi:10.3389/fpls.2022.1015658)
Supplement: Supplementary file 2 [file DataSheet_2.pdf]

Figure S1. Diagram of apple shape classifications, modified from Watkins and Smith (1997).

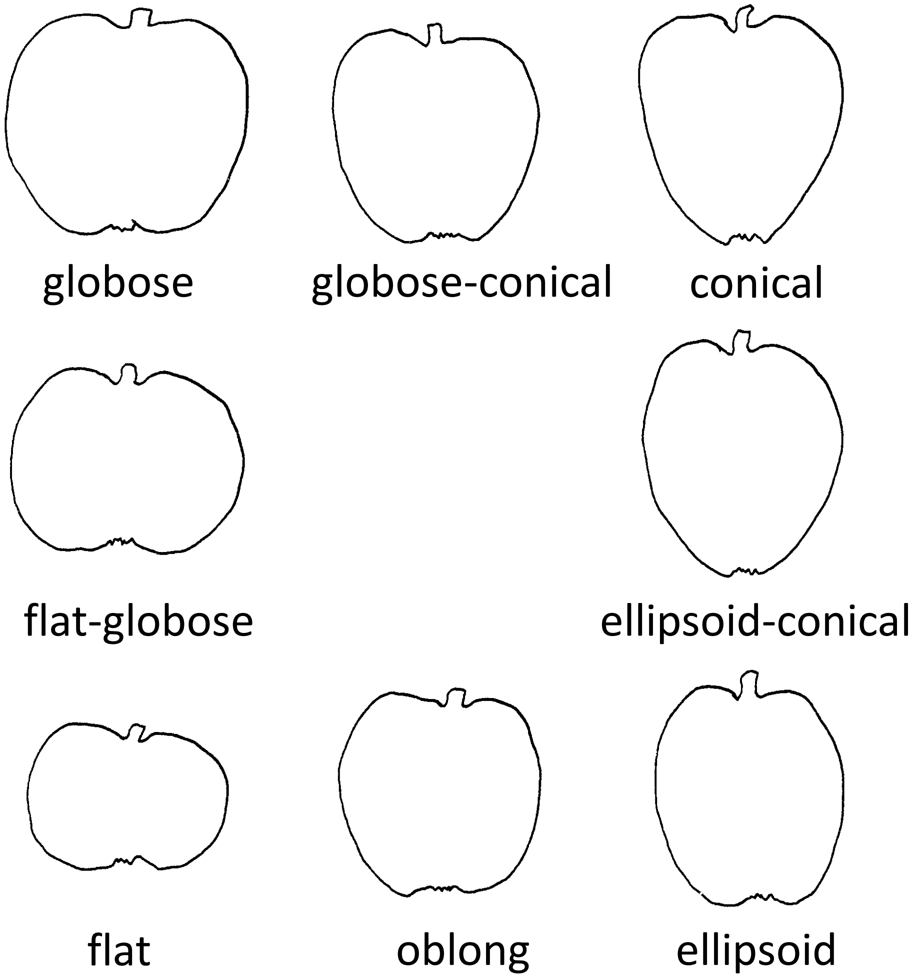

Figure S2. Clustering results for principal component analysis of NPGS accessions labeled as *M. sieversii* (n=157), *M. orientalis* (n=26), and *M. sylvestris* (n=9) to confirm species status. Outliers from the pure species groups were accessions that were identified as being hybrids or admixed. Proportions of variance explained by the first two principal components are shown on the x and y axes.

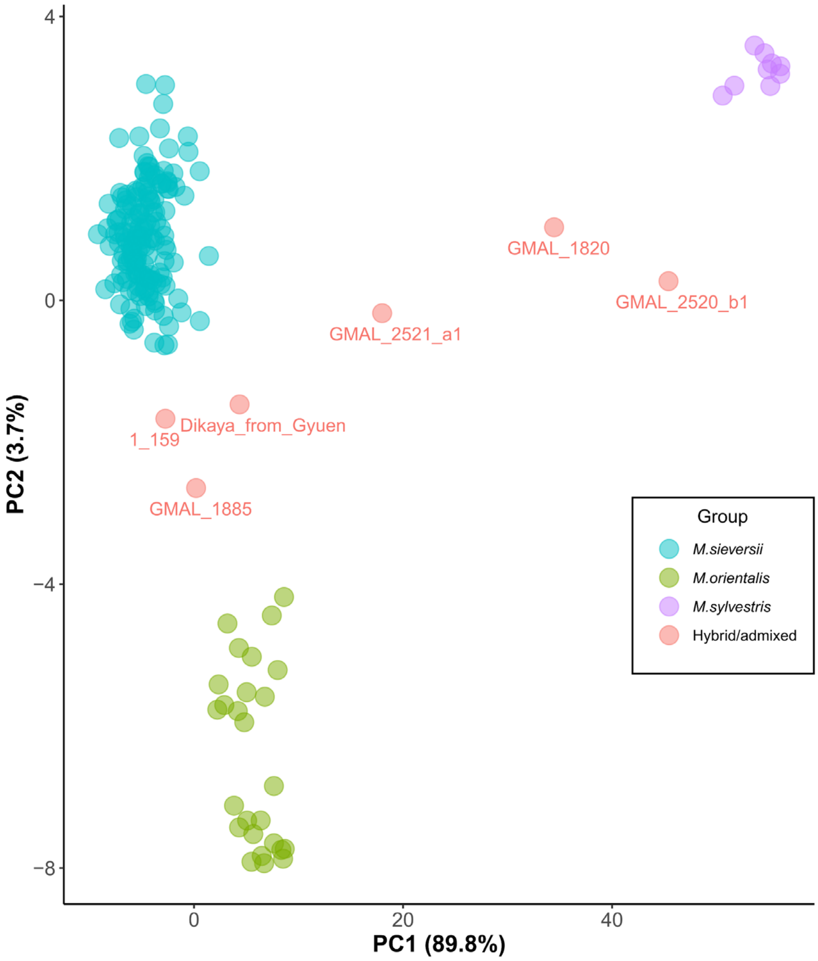

Figure S3. Examples of pure *Malus sieversii accessions* representing the observed fruit shapes.

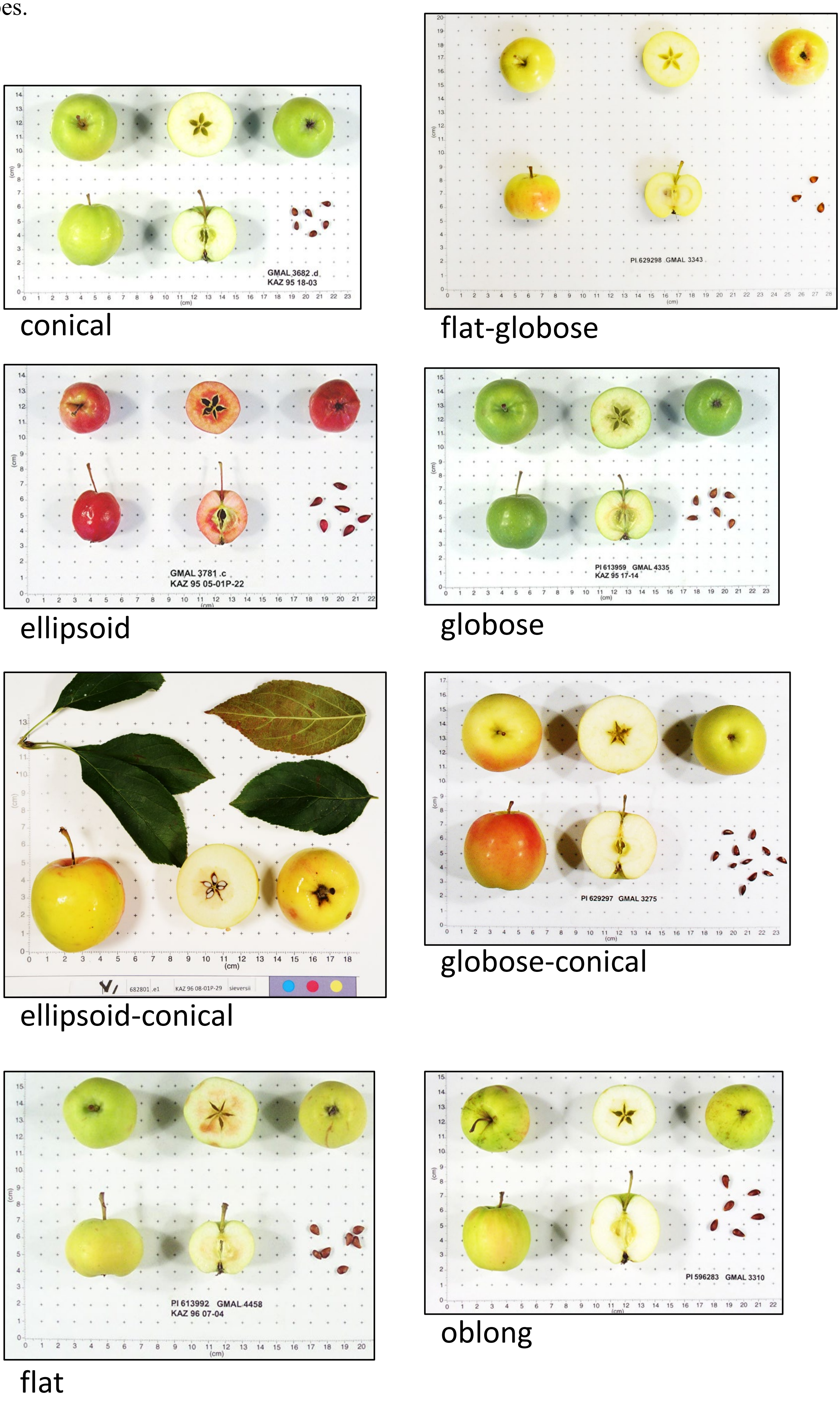

Figure S4. Examples of pure *Malus orientalis* accessions representing the observed fruit shapes.

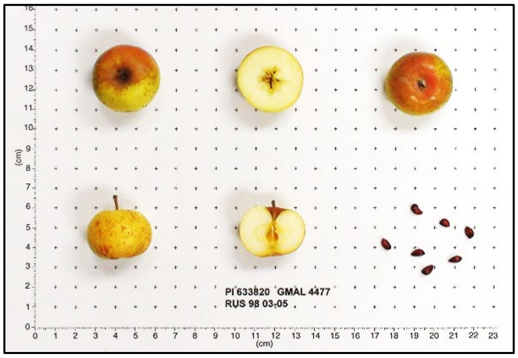

flat

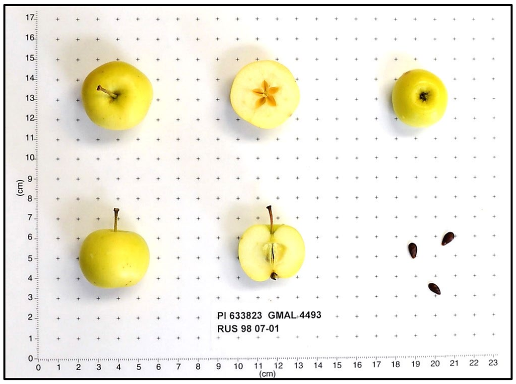

flat-globose

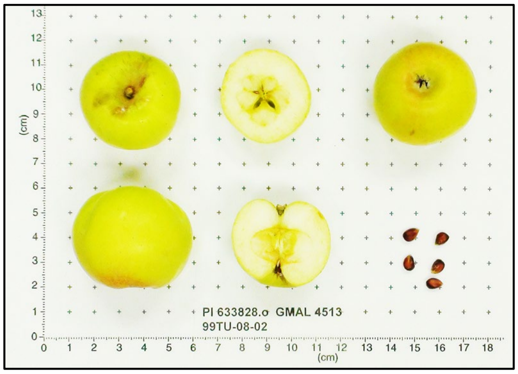

globose

Figure S5. Examples of pure *Malus sylvestris* accessions representing the observed fruit shapes.

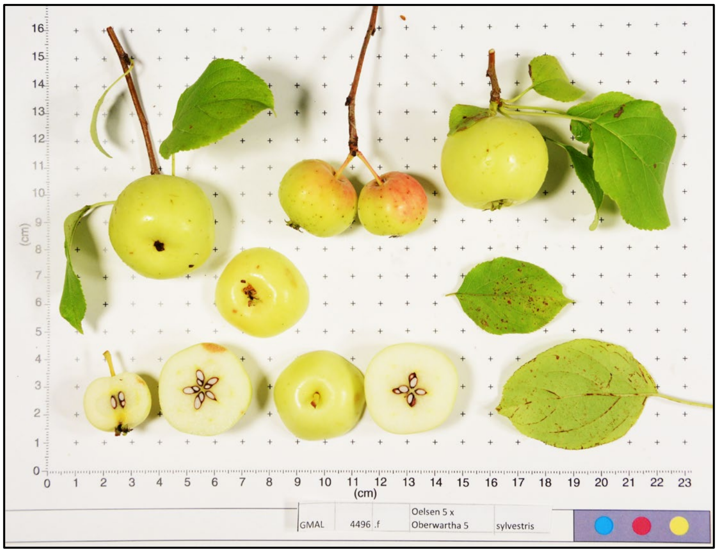

flat-globose

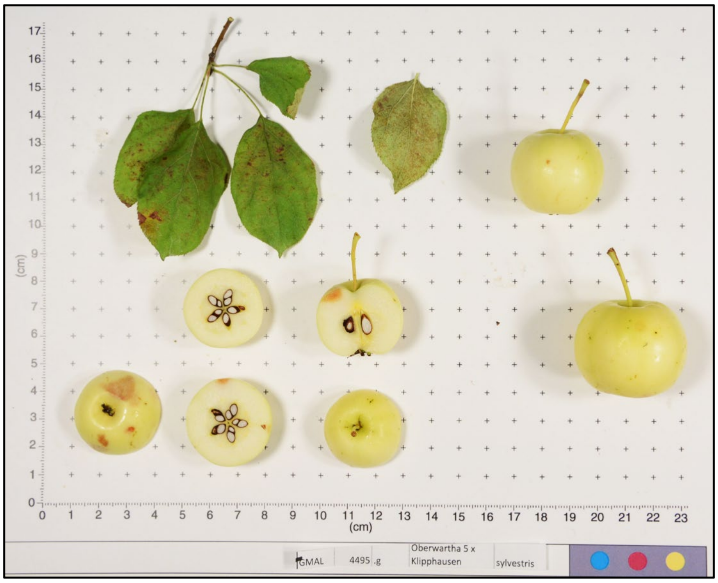

globose
